# Posters, june 7

**DOI:** 10.3402/ejpt.v4i0.22652

**Published:** 2013-08-29

## XIII ESTSS CONFERENCE: “Trauma and its clinical pathways: PTSD and beyond”, Bologna, June 2013   POSTERS, JUNE 7

## Impact of trauma on communities

### Prevalence rate of post-traumatic stress disorders (PTSD) and other psychological disorders among Saudi firefighters

#### Mohammed Alghamd^1^, Nigel Hunt^2^ and Shirley Thomas^3^ ^1^Clinical Psychology, Division of Psychiatry and Applied Psychology, School of Medicine, University of Nottingham, Nottingham, UK; ^2^Division of Psychiatry and Applied Psychology, School of Medicine, University of Nottingham, Nottingham, UK; ^3^Rehabilitation Psychology, Division of Rehabilitation & Ageing, School of Medicine, University of Nottingham, Nottingham, UK

*Background*: Firefighters have a high probability of being exposed to a variety of traumatic events. Potentially traumatic events can occur during a single rescue such as: providing aid to seriously injured or helpless victims. Moreover, firefighters who are injured in the line of duty may have to retire as a consequence of their injury. The psychological cost of this exposure may increase the risk of long-term problems, such as post-traumatic stress disorder (PTSD) symptoms, depression, and anxiety. *Objective*: The purpose of this study was to investigate the prevalence of PTSD symptoms, depression, anxiety, and assess related variables such as coping strategies and social support among Saudi firefighters. *Method*: Two hundred firefighters completed the Fire-fighter Trauma History Screen (FTHS) to measure the number of traumatic events, Screen for Post-traumatic Stress Symptoms (SPTSS) scale to assess the prevalence of PTSD symptoms, Hospital Anxiety and Depression Scales (HADS) to assess depression and anxiety, Brief Cope (BC) scale to measure coping strategies used, and Social Support scale was used to evaluate the firefighter's support received. *Results*: The results showed that 84% (169/200) of firefighters were exposed to at least one traumatic event. The result presented that 57% (96/169) of exposure firefighters fully met the DSM-IV criteria for PTSD with high levels of depression and anxiety; 39% (66/169) partially met the PTSD criteria. However, only 4% participants have not met the PTSD criteria. The results also revealed that adaptive coping strategies and higher perceived social support was associated with lower levels of PTSD. *Conclusion*: The high prevalence rate of PTSD related to the type and severity of the traumatic events and years of experience in the job. Accordingly, many firefighters were severely affected by their experiences, and we should be developing methods to help them.

